# Perspective Change and Personality State Variability: An Argument for the Role of Self-Awareness and an Outlook on Bidirectionality (Commentary on Wundrack et al., 2018)

**DOI:** 10.3390/jintelligence7020010

**Published:** 2019-04-17

**Authors:** Emanuel Jauk, Philipp Kanske

**Affiliations:** 1 Clinical Psychology and Behavioral Neuroscience, Faculty of Psychology, Technische Universität Dresden, 01187 Dresden, Germany; 2 Max Planck Institute for Human Cognitive and Brain Sciences, 04103 Leipzig, Germany

**Keywords:** perspective-taking, theory of mind, personality states, intraindividual variability, self-awareness

## Abstract

In a recent article, Wundrack et al. (2018) put forward an elaborate and intriguing hypothesis on enhanced perspective-taking (Theory of Mind) ability as a consequence of higher personality state variability. While there is evidence in favor of this hypothesis, the clinical examples of bipolar disorder and borderline personality disorder, as highlighted by the authors, demonstrate that a high state variability can also be accompanied by a lower perspective-taking ability (as commonly observed in these disorders). We suggest that only those state changes which are initiated on a voluntary basis and are accompanied by self-awareness go along with a higher perspective-taking ability. Introducing self-awareness as a moderating factor might help explain seemingly conflicting findings related to the hypothesis proposed in the target article. Moreover, we argue that the direction of causality proposed in the target article is not the only conceivable one, and perspective-taking ability could also be a cause, not just a consequence, of personality state variability. Finally, we provide suggestions on how these hypotheses could be tested in future studies.

Wundrack et al. [1] put forward an intriguing hypothesis on gains in perspective-taking ability as a consequence of state changes in personality dimensions. They argued that experiencing a greater variety of different personality states—in terms of the varying expressions of affective, cognitive, and behavioral patterns associated with certain personality dimensions—leads to a greater likelihood of ego-dispersion, i.e., a reduction of egocentric bias, and perspective pooling, i.e., adjustment to someone else’s perspective.

This hypothesis is not only conceptually well-founded and appealing, but also supported by recent empirical evidence. Böckler et al. [2] reported that a three-month “inner parts” intervention increases perspective-taking (used here synonymously with Theory of Mind (ToM) for training effects, see also [3,4]). During the intervention, participants were asked to identify their inner parts, which can be thought of as “relatively discrete subpersonalities which are each characterized by specific affective, cognitive, and behavioral patterns” [2] (p. 199). Examples for these parts are for instance the inner critic or the inner protector [5], which can be thought of as variances in the personality dimension of antagonism/agreeableness. Interestingly, the number of inner parts that were identified by participants in the course of the training directly related to increases in ToM performance in terms of understanding others mental states. This can be interpreted as evidence for the mechanism proposed in the target article, as it shows that experiencing variation in personality dimensions was accompanied by increases in ToM performance. Though the study design focused on inner state changes rather than state changes evoked by different external situations, the associated experiences are real in that they go along with different patterns of affect, cognition, and behavior just as state changes in the outer world. It is noteworthy that state changes were performed on a voluntary, conscious basis in this study, as participants actively tried to switch and take the perspectives of their different personality parts.

As also noted in the target article, not all state variability is accompanied by increased ToM performance. Specifically, the clinical examples of bipolar disorder and borderline personality disorder highlighted by the authors show that high state variability—as evident in bipolar disorder and borderline personality disorder—can also be accompanied by reduced, not heightened ToM (for meta-analyses, see [6,7]). Here, it needs to be noted that for the latter example, borderline personality disorder, the effects are more heterogeneous than for bipolar disorder, and might depend on the used ToM measure. Studies using emotion recognition ability as a proxy for ToM find an intact [8] or even elevated ability [7,9] in borderline personality disorder. However, individuals with borderline personality disorder display lower ToM in complex, real-life ToM tasks [7,8,10], which arguably have the highest ecological validity. ToM deficits in borderline personality disorder can, depending on emotional arousal, result from overmentalizing (excessive, but incorrect mentalizing) [10,11,12] or also under- or nonmentalizing under high arousal [13]. In bipolar disorder, undermentalizing seems to be the primary cause of reduced ToM performance [14].

Given that both bipolar disorder and borderline personality disorder can be viewed as extremes of general, non-pathological variation [15,16] (see also [17]), we believe a crucial and central question is how more state variability can go along with a lower perspective-taking ability. These examples partially challenge the hypothesis proposed in the target article as they point to the necessity of introducing moderating factors under which state changes lead to ToM enhancement.

We suggest that whether or not state variability is accompanied by increased perspective-taking might depend on whether or not state changes are initiated on a voluntary and conscious basis. State variability is commonly not experienced as voluntary by patients with bipolar or borderline personality disorder. On the contrary, these patients experience being at the mercy of inner or outer forces that determine their patterns of experience and behavior, which manifests in reduced cognitive control [18,19,20] and emotional regulation [21,22] as well as increased impulsivity [23] in these disorders. For bipolar disorder, reduced cognitive control is at the same time discussed as a potential mechanism of reduced ToM performance [24].

Volition in itself, however, cannot explain why some state changes are accompanied by ToM increases whereas others are not. Involuntary state changes could serve ego-dispersion and perspective pooling as well. Voluntary state changes may, however, be accompanied by *self-awareness*, which might be crucial to the deliberate experience of different personality states. Self-awareness, defined as *becoming the object of one’s own attention* [25], can vary between different states, but can also be habitually high or low, therefore becoming a trait itself (also referred to as private and public self-consciousness) [26]. While habitually high self-awareness is not associated per se with desirable or undesirable psychological characteristics (and can take the form of both, reflection or rumination concerning the private self [27] or acquisitive and protective self-monitoring concerning the public self [28,29,30]), habitually low self-awareness might be indicative of disorders characterized by reduced personality functioning (i.e., reduced self and other-related mental functioning [31]).

Indeed, different aspects of self-awareness are known to be reduced in bipolar disorder [32] and borderline personality disorder [33,34]. The lower degree of self-awareness during state changes in these disorders might explain why these changes may not be accompanied by ego-dispersion and perspective pooling—the processes hypothesized to underlie ToM increases—in these cases. State changes that are of a primarily *reflexive* (i.e., automatic, low awareness), not a *reflective* (voluntary, high awareness) nature, might be of little benefit to ego-dispersion or perspective pooling, as they do not include conscious self-insights such as “right now, I am feeling and behaving differently than just before”. We think this is well in line with the author’s (positive) definition of ego-dispersion as “the general awareness of the transiency of your own perspective” [1] (p. 50).

If we now integrate the previously mentioned findings of Böckler and colleagues [2] with the idea that only state changes accompanied by self-awareness go along with ego-dispersion and perspective pooling—which are hypothesized to underlie ToM enhancement—it could be speculated that the direction of causality assumed by Wundrack et al. [1] is not the only possible direction. Indeed, taking the idea of inner perspective changes one step further, it could be hypothesized that the perspective-taking ability cannot only be a consequence but also an antecedent of higher reflective (voluntary and self-aware) state variability. It might be the case that perspective-taking ability acts as a facilitator of switching between different experiential and behavioral patterns, which then manifests in terms of personality state changes. The capacity to mentally simulate different personality states might thus also precede changes in observable behavior. 

The idea that perspective-taking can lead to state changes is supported by recent evidence from Gilead et al. [35]. In this study, participants were instructed to take the perspective of either an emotionally “sensitive” or “tough” individual. Participants subsequent emotional and neural responses to affective pictures were in line with the emotional state of the imagined individual, that is, reduced reactivity if participants took the perspective of the “tough” person. This suggests that voluntary perspective-taking, in terms of a simulation of others mental states, can lead to an alteration in their own experiences in the direction of these states. The process is very close if not identical to perspective pooling as proposed in the target article, with the exception that the causal direction is reversed in this case. The result of this process is, in this case, dispersed egocentricity. Interestingly, neuroscience research also shows that metacognitive ability in ToM (i.e., the evaluation of one’s own ToM performance) is associated with activity in parts of the ToM network itself, which suggests that the awareness of perspective-taking processes may be an intrinsic and vital part of perspective-taking ability [36].

If perspective-taking can also be a cause, not just a consequence, of behavioral variability, this might also explain the seemingly paradoxical findings for patients with bipolar or borderline personality disorder. As perspective-taking ability is, as discussed above, typically lower in these individuals, they might experience little control over different personality states, leaving them with high variability at low self-awareness. In line with this, therapeutic interventions for borderline patients were designed specifically to increase perspective-taking as part of general mentalizing ability [37].

For future studies investigating the mutual relations between personality state variability and perspective-taking ability, two suggestions may be derived from the presented considerations. First, self-awareness could be considered as a moderating intra-or interindividual (depending on the research design; see below) difference variable. Intraindividual differences could, for instance, be assessed using the situational self-awareness scale [38], interindividual differences could be assessed using items in the trait of self-awareness in terms of *reflection* [27] or also via conceptually related broader traits such as mindfulness (for a review of scales, see [39]). As noted in the target article, self-monitoring in terms of an awareness of the public self might also play a role in controlled personality state variability [1]. From the considerations presented here, it follows that the relation between personality state variability and ToM performance should be stronger in individuals who display high self-awareness. 

This, however, does not speak to the causality of effects, which brings us to the second point. Since state variability in real-life settings can hardly be experimentally varied to investigate causal relationships (though ToM could be experimentally varied by means of training, as noted in the target article), longitudinal, quasi-experimental studies could investigate cross-lagged effects to estimate the relative mutual influences among personality state variability and perspective-taking. To this end, perspective-taking could be assessed using naturalistic, complex ToM tasks, as discussed above (though such research is complicated by the fact that these cannot be repeated at will, up to four measurements were already used in prior studies [3]), social inference tasks [40], as proposed in the target article, or self-reports of perspective-taking in real situations (as in empathy research [41]). These could be combined with subjective and objective assessments of personality states (including situational self-awareness) using Ecological Momentary Assessment. We wish to emphasize that the suggestions provided here can be seen as possible extensions to the research program proposed by Wundrack et al. [1], which might be helpful in broadening the scope of the proposed hypothesis. Still, the research outlined in the target article appears to be an important first step, from our point of view.

Taken together, the hypothesis proposed in the article by Wundrack et al. [1] is a highly innovative approach to explain interindividual variability in perspective-taking capabilities. It not only possesses face validity, but is also supported by first empirical evidence. We propose here that self-awareness might be an important moderating factor that determines whether or not higher personality state variability might go along with an increased perspective-taking ability and the direction of causality implied might not be the only conceivable one. Indeed, both causal pathways seem plausible based on past research on perspective-taking and behavioral variability. Longitudinal studies employing Ecological Momentary Assessment could provide insights into the causal nature of the hypothesized relations and their putative mutual interplay. Varying degrees of volition and associated self-awareness might moderate the association between state changes and perspective-taking.
